# Spatiotemporal Differences in Gene Expression Between Motor and Sensory Autografts and Their Effect on Femoral Nerve Regeneration in the Rat

**DOI:** 10.3389/fncel.2019.00182

**Published:** 2019-05-08

**Authors:** David Hercher, Markus Kerbl, Christina M. A. P. Schuh, Johannes Heinzel, László Gal, Michaela Stainer, Robert Schmidhammer, Thomas Hausner, Heinz Redl, Antal Nógrádi, Ara Hacobian

**Affiliations:** ^1^Ludwig Boltzmann Institute for Experimental and Clinical Traumatology, Vienna, Austria; ^2^Austrian Cluster for Tissue Regeneration, Vienna, Austria; ^3^Centro de Medicina Regenerativa, Facultad de Medicina Clínica Alemana-Universidad del Desarrollo, Santiago, Chile; ^4^Department of Anatomy, Histology and Embryology, University of Szeged, Szeged, Hungary

**Keywords:** femoral nerve, Schwann cell, phenotype, gene expression, neurotrophic factor, cell adhesion molecule, peripheral nerve regeneration

## Abstract

To improve the outcome after autologous nerve grafting in the clinic, it is important to understand the limiting variables such as distinct phenotypes of motor and sensory Schwann cells. This study investigated the properties of phenotypically different autografts in a 6 mm femoral nerve defect model in the rat, where the respective femoral branches distally of the inguinal bifurcation served as homotopic, or heterotopic autografts. Axonal regeneration and target reinnervation was analyzed by gait analysis, electrophysiology, and wet muscle mass analysis. We evaluated regeneration-associated gene expression between 5 days and 10 weeks after repair, in the autografts as well as the proximal, and distal segments of the femoral nerve using qRT-PCR. Furthermore we investigated expression patterns of phenotypically pure ventral and dorsal roots. We identified highly significant differences in gene expression of a variety of regeneration-associated genes along the central – peripheral axis in healthy femoral nerves. Phenotypically mismatched grafting resulted in altered spatiotemporal expression of neurotrophic factor BDNF, GDNF receptor GFRα1, cell adhesion molecules Cadm3, Cadm4, L1CAM, and proliferation associated Ki67. Although significantly higher quadriceps muscle mass following homotopic nerve grafting was measured, we did not observe differences in gait analysis, and electrophysiological parameters between treatment paradigms. Our study provides evidence for phenotypic commitment of autologous nerve grafts after injury and gives a conclusive overview of temporal expression of several important regeneration-associated genes after repair with sensory or motor graft.

## Introduction

Injuries to the upper extremities are amongst the most common work-, sports- and traffic related injuries. These injuries may result in peripheral nerve damages with loss of nerve continuity and subsequent loss of motor and sensory function. They require long rehabilitation phases and have a major impact on the quality of life of the patient. The clinical gold standard to bridge a nerve injury with segmental loss is the autologous nerve transplant. In clinical cases, a sensory nerve is harvested and used as a transplant to bridge the defect in order to restore essential motor functions. However, functional outcome after nerve grafting is very often not satisfactory, especially after nerve injury with profound tissue loss (Meek et al., 2005; Secer et al., 2008; Yang et al., 2011). A reason could be the presence of phenotypically mismatched Schwann cells in the purely sensory nerve grafts. Several groups have provided evidence that there are phenotypical differences between Schwann cells from sensory and motor nerves regarding the expression of neurotrophic factors and their receptors (Höke et al., 2006; Chu et al., 2008; He et al., 2012b; Jesuraj et al., 2012, 2014). Furthermore, sensory and motor branches of the femoral nerve differ in architecture, thereby influencing regeneration of axons after injury (Moradzadeh et al., 2008). The majority of publications investigating the effect of sensory nerve grafts suggests a negative impact of sensory phenotype on motor axon regeneration when compared to a motor graft (Sulaiman et al., 2002; Brenner et al., 2006; Abdullah et al., 2013). However, a comprehensive study investigating the effect of phenotypically different grafting including functional outcome, gene expression patterns and histology has not been published yet. We hypothesized that there are differences in the gene expression patterns in sensory Schwann cells when compared to Schwann cells derived from motor nerves and that this phenotypical commitment has an effect on the regenerative capacity of axons. First, we investigated the baseline gene expression levels along the central-peripheral axis in uninjured femoral nerves with emphasis on phenotypical differences. Second, we determined the influence of a phenotypically different environment on the regenerative capacity of motor and sensory axons, thereby establishing a characteristic expression profile along the phenotypical mixed, motor (carrying afferent and efferent fibers from/to the muscle) and purely sensory nerves. For this study we used a preclinical *in vivo* model, which reflects the clinical situations (i.e., the use of autologous sensory nerve graft to support axonal regeneration and restore motor function). Therefore we performed homotopic as well as heterotopic nerve autografting in a femoral nerve defect model. Taken together, this study evaluated the spatiotemporal expression of key regeneration-associated genes and their influence on functional reinnervation. We aim to add insight into regenerative processes in phenotypically mismatched environments to allow future development of strategies to improve functional outcome after nerve grafting surgeries.

## Materials and Methods

### Experimental Model

**FIGURE 1:**
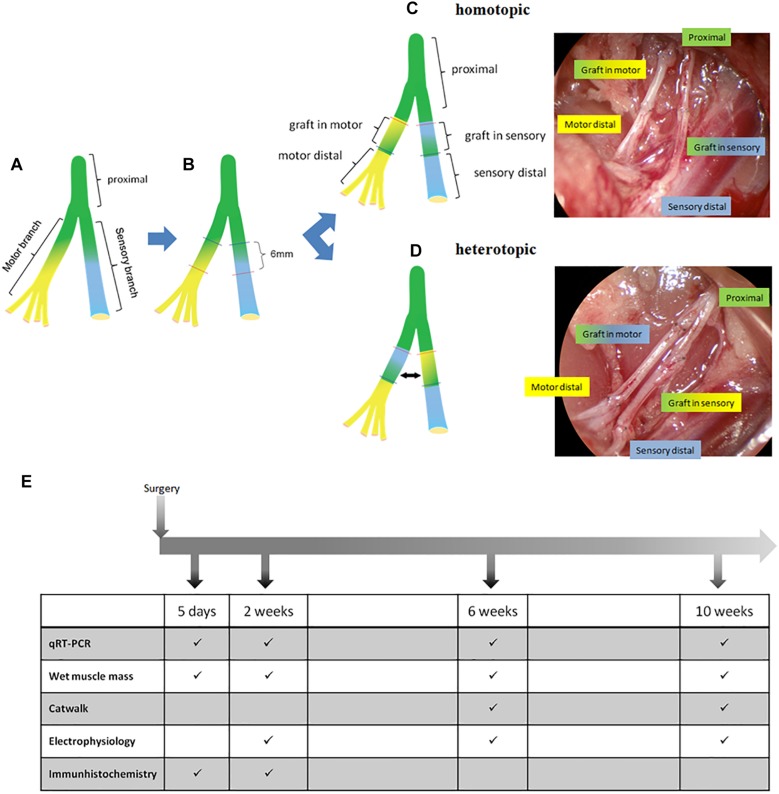
Overview of surgical procedure and experimental timeline. **(A)** Femoral nerve at the bifurcation into motor and sensory branch. **(B)** Transection sites on both branches resulting in a 6 mm gap and 6 mm graft. **(C)** Homotopic (excised motor segment is turned by 180° and used to bridge the motor defect, the excised cutaneous segment is used to bridge the cutaneous defect). **(D)** Heterotopic autologous nerve transplantation in both branches of the femoral nerve with intra-operative result after repair. **(E)** timetable of follow up and analyses.

To analyze the spatiotemporal gene expression changes during nerve regeneration the establishment of an appropriate animal model was mandatory for this study. We adapted the rat femoral nerve model, described earlier to the present study, as it bifurcates into a mixed motor, and a purely sensory branch at the inguinal region (Brushart, 1990; Jesuraj et al., 2012; He et al., 2016). A power analysis was performed using StatMate 2.0 (GraphPad) in order to evaluate appropriate sample size and power. A total of 99 male *Sprague Dawley* rats (Charles River, Sulzfeld, Germany) weighing 300–350 g were randomly assigned to treatment groups. Group sizes were as follows: gene expression analysis (*n* = 8–10), histological evaluation (*n* = 3–5), and retrograde labeling (*n* = 2–3). Animals were subdivided in 4 different observation time groups: 5 days, 2 weeks, 6 and 10 weeks (Figure 1E and Table 1).

**TABLE 1:** Nerve segments used for qRT-PCR.

**Excised nerve samples for qRT-PCR analysis**	
**Healthy contralateral**	**Injured ipsilateral**
Dorsal roots (DR) L2 and L3	
Ventral roots (VR) L2 and L3	
Femoral nerve proximal of bifurcation	Femoral nerve proximal of bifurcation
Cutaneous branch of femoral nerve	Graft in cutaneous branch
	Cutaneous branch distal of graft
Motor branch of femoral nerve	Graft in motor branch
	Motor branch distal of graft

*Baseline expression was evaluated using the contralateral, healthy nerves. Effect of injury and graft phenotype was evaluated by comparing the nerve segments from the injured side to their contralateral counter part in the same animal.*

Animals were kept pairwise in appropriate cages according to internal standard operating procedures, including food (Sniff, Soest, Germany), and water *ad libitum*. Animals were allowed to accustomize for 7 days prior to any experimental handling.

### Surgical Procedure

All procedures were performed under aseptical conditions according to Austrian law and the guide for the care and use of laboratory animals, and were approved by the City Government of Vienna (Animal use permit: MA58 149539/2012/5). Under general anesthesia (110 mg/kg Ketamin, 12 mg/kg Xylazin; inhalation of 1–2 Vol% Isofluran-Oxygen mix) and analgesia (1× daily 1.25 mg/kg Butorphanol s.c. and 1× daily 0.15 mg/kg Meloxicam for 4 days starting on the day of surgery) a longitudinal 3–4 cm groin incision was applied in order to expose the right femoral neurovascular bundle. Blunt exploration was performed until the bifurcation of the femoral nerve was exposed. The exposed motor and sensory branches were sharply transected distal to the bifurcation resulting in a 6 mm gap and a 6 mm graft of each branch, respectively (Figure 1B). Elastic retraction of nerves was negligble and tension-free suturing was carried out. According to the assigned treatment group the gaps were repaired with either a homotopic graft (motor graft in motor branch and sensory graft in sensory branch) (Figure 1C) or a heterotopic graft (motor graft in sensory branch and sensory graft in motor branch) (Figure 1D) using 2 epineural sutures per coaptation site (Ethilon 11-0; Ethicon, Somerville, NJ, United States). Postoperative analgesia was given once a day for 4 days.

### Catwalk Automated Gait Analysis

Functional analysis was performed with the CatWalk XT (Version 10.6) automated gait analysis system (Noldus Information System, Wageningen, Netherlands). (Pena and Baron, 1988; Hamers et al., 2001; Vrinten and Hamers, 2003; Koopmans et al., 2007; Bozkurt et al., 2008, 2011, 2012; Hausner et al., 2012; Huang et al., 2012; Li et al., 2014; Kappos et al., 2017).

Animals were pre-trained to use the CatWalk daily, for 1 week prior to surgery. Data was collected according to the recommendations made in the literature (Deumens et al., 2014). Parameters assessed were print area, swing time, and duty cycle.

Data was obtained from 37 animals in total, which were subdivided into the following groups:

Homotopic nerve graft (*n* = 19, observed 6 weeks: *n* = 9, observed 10 weeks: *n* = 10).

Heterotopic nerve graft (*n* = 18, observed 6 weeks: *n* = 10, observed 10 weeks: *n* = 8).

We collected 3 runs per trial at baseline and from then every week, ending data collection either 6 weeks (*n* = 19) or 10 weeks (*n* = 18) after surgery.

Since strong emphasis has been placed on crossing time or crossing speed as crucial factors to control for proper analysis of data we collected 3 runs per trial, within the exact same velocity ranges as defined by Koopmans et al. (2007). Additionally, only those runs were recorded which contained at least 3 step cycles per paw.

Each of the three parameters (Print Area, Swing Time and Duty Cycle) was assessed for both hind paws and for each one a ratio was calculated by dividing the right side’s value (transection) by the left side’s (control). This ratio (Print Area ratio, Swing Time ratio, Duty Cycle ratio) was then compared to the ratio at baseline for each postoperative time point and the result given in percent.

### Electrophysiology and Quadriceps Muscle Mass

In order to evaluate successful reinnervation of the quadriceps muscle, compound muscle action potential (CMAP), and peak amplitude of voltage signal was measured at the end of the observation time at 2, 6, and 10 weeks. The femoral nerve was explored and for stimulation of the motor branch a bipolar stimulation electrode was placed proximal to the bifurcation using a micro manipulator. Two needle electrodes were placed into the quadriceps muscle approximately 10 mm apart for recording, whereas the grounding electrode was placed in the surrounding tissue. A Neuromax EMG device (Natus, WI, United States) was used for stimulation and recording. The contralateral healthy femoral nerve and quadriceps muscle served as an internal control. Core temperature of the animal was measured rectally and used for normalization.

#### (Muscle) Mass Evaluation

At all endpoints quadriceps muscles were excised bilaterally and weighed to evaluate atrophy and regeneration. Muscle weight was set in to relation of total weight of the animal.

### (Immuno)-Histological Evaluation

For immunohistological evaluation, femoral nerves were harvested 5 days and 2 weeks after surgery and fixated in 4% buffered formaldehyde (VWR, Radnor, PA, United States) overnight. Subsequently, nerves were washed (distilled water) and transferred into 30% sucrose in phosphate buffered saline (PBS). 25 μm thick longitudinal sections were cut using a cryostat and mounted on gelatine-coated glass slides.

In order to evaluate axonal reinnervation, sections were blocked with 5% skim milk and subsequently stained with anti-Neurofilament (rabbit monoclonal; Abcam, United Kingdom) overnight at 4°C. After washing with PBS, sections were incubated with secondary antibody (anti-rabbit Alexa Fluor 488 Life Technologies, MA, United States) mounted for microscopical evaluation using glycerol.

Cresyl violet staining was performed as follows: cryosections were rinsed in distilled water and stained with 1% w/v Cresyl Violet solution pH 4 (Sigma-Aldrich) for 5–10 min. Samples were mounted with DPX mounting media (Sigma-Aldrich).

### Quantitative Real Time Reverse Transcription Polymerase Chain Reaction (qRT-PCR)

For gene expression analysis, nerve fragments were harvested from all observation time groups 5 days, 2, 6, and 10 weeks (*n* = 8–10), according to Table 1. Samples were immediately snap frozen in liquid N_2_ and stored on −80°C. RNA was isolated using peqGOLD TriFast according to manufacturer’s instructions (VWR, United States) and used for subsequent cDNA synthesis (EasyScript^TM^ RTase, ABM, CAN). Analysis of mRNA levels were performed by qRT-PCR using 18 ng cDNA, primer according to Table 2, and KAPA SYBR (KAPA SYBR, Peqlab/PerfeCTa SYBR, VWR, United States) on a Bio-Rad CFX 96 cycler (Bio-Rad, CA, United States) (for detailled protocol see Supplementary Data).

**TABLE 2:** Primer sequence, annealing- and melting temperature, and detected transcript variants.

**Target name**	**Primer sequence**	**Annealing temp. (exp)**	**Melting temp. (exp)**	**Transcript variants**
**BDNF**	s: TGA G AGA C G CAC AGG A	59.0°C	77.5°C	1-10 X1
NM_012513.4	as: AGA GGT A GTG TAG G GGA C			
**GDNF**	s: G TAG G GCT C G TGA C	60.0°C	82.0°C	X1–X4
NM_019139.1	as: CCA GGG TCA GAT ACA TCC AC			
**L1CAM** (He et al., 2012a)	s: GCCTCAGCCTCTATGTG	60.0°C	80.0°C	X1–X4
NM_017345.1	as: GCCAGTGCCATTAGTCTTC			
**HPRT**	s: AGT CCC AGC GTC GTG ATT AG	63.5°C	78.0°C	HPRT, HPRT1
NM_012583.2	as: TGG CCT CCC ATC TCC TTC AT			
**Fibronectin** (Yoshida et al., 2002)	s: GTGGCTGCCTTCCTTCTC	58.0°C	80.0°C	X1–X10
NM_019143.2	as: GTGGGTTGCACCTTCT			
**Ankrd27** (Gambarotta et al., 2014)	s: CCCAGGATCCGAGAGGTGCTGTC	62.0°C	79.0°C	X1, X6
NM_001271264.1	as: CAGAGCCATATGGACTTCAGGGGG			
**GFAP** (Tanga et al., 2004)	s: TGG CCA CCA GTA ACA TGC	62.0°C	83.0°C	GFAP
NM_017009.2	as: CAGTTGGCGGCGATAGTCAT			
**TrkB** (Kondo et al., 2010)	s: CACACACAGGGCTCCTTA	63.0°C	80.0°C	X1–X5
NM_012731.2	as: AGTGGTGGTCTGAGGTTGG			
**Itgα5**	s: GTTTACACATGCCCTCTCAC	59.0°C	80.0°C	Itgα5
NM_001108118.1	as: GATTCCCTTTACAGCTCCA			

**Itgß1**	s: A CAG T C AGA ACA GTC C	56.0°C	81.0°C	X1
NM_017022.2	as: AGG ATC G T CTC ACA ATG G			
**MKi-67**	s: ACT GTA T T ATT GCC TGT GCT	60.0°C	78.0°C	X1
NM_001271366.1	as: CCC ACC A CAT TTA C TGC T			
**GFRα1**	s: CTG TCT TTC TGA TAG TGA TTT CGG	56.0°C	83.5°C	X1,X2
NM_012959.1	as: GTG A CTG CTT CTC C GG			
**Cadm3**	s: CTCTCTGTCCGACCCT	60.0°C	80.5°C	X1
NM_001047103.1	as: TTTGTGCCGGATCATAGTG			
**Cadm4**	s: CAG TAT GAT GGG TCT ATA GTC GT	64.0°C	85.5°C	Cadm4
NM_001047107.1	as: GGA GCC ACT G ACA GTG AG			
**LIFR**	s: CATCTACTGGGCCTTTACC	59.0°C	78.5°C	X1–X3
NM_031048.1	as: TCTCTGCTTTGTGTTGTGGA			
**p75**	s: CTG T GCG G AGA TCC CT	60,0°C	85,0°C	X1,X2
NM_012610.2	as: G ATG GAG C TAG ACA GGA			
**HSP70**	s: GGTGCTGATCCAGGTGTACGAGG	55.0°C	85.0°C	Hspa1a, Hspa1b, Hspa1l
NM_212546.4, NM_031971.2, NM_212504.1	as: GATGTCGGGTCACCTCGATCTGG			
**ß-Actin** (Kondo et al., 2010)	s: CCTGTATGCCTCTGGTCGTA	55.0°C	85.5°C	Actb
NM_031144.3	as: CCATCTCTTGCTCGGTCT			

We focused our investigation on three groups of targets: First, neurotrophins and their receptors, as they play crucial roles in regeneration and maintenance of the peripheral nervous system. Second, cell adhesion molecules, with special emphasis on Schwann cell – axon interactions. Third, a group of genes, which give us information about the cell status, again with emphasis on Schwann cells.

We tested several reference genes including beta-actin (ß-Actin) and ankyrin repeat domain-containing protein 27 (Ankrd27) (Gambarotta et al., 2014) in a subset of samples, however, we did not detect a significant influence of central-peripheral axis and phenotype as well as injury on the expression levels between HPRT and others (Supplementary Figure 1). Data were normalized to Hypoxanthine-guanine phosphoribosyltransferase (HPRT) mRNA level in the same sample.

### Statistical Analysis

Statistical analysis was performed using GraphPad Prism 6.0 (GraphPad Software Inc., San Diego, CA, United States). The data was tested for normal distribution using the Kolmogorov–Smirnov test. Data was statistically analyzed using either Student’s *t*-test (two groups to compare), multiple *t*-tests using Holm-Sidak correction for multiple comparison or one-way ANOVA, and Tukey’s multiple comparison *post hoc* test. To compare various experimental groups among different time points, two-way ANOVA with Tukey’s multiple comparison *post hoc* tests was used. Results are expressed as means with standard error of the mean (SEM). *P*-value of <0.05 was considered as statistically significant.

## Results

### Functional Reinnervation Is Comparable After Homotopic and Heterotopic Autografting

#### Gait Analysis Is a Feasible but Limited Functional Testing Method in Femoral Nerve Defect Models

**FIGURE 2:**
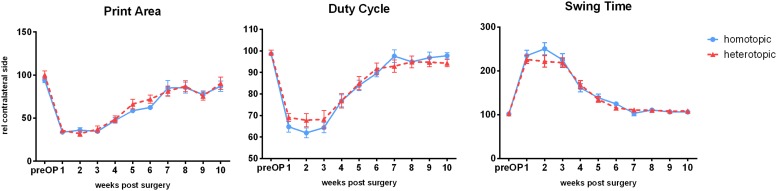
Weekly Catwalk automated gait analysis after homotopic or heterotopic autografting: All three parameters showed injury-dependent changes and recovered over the observation period of 10 weeks *ad integrum.* No significant differences between treatment groups were observed. Data is depicted as means ± SEM, at least 3 comparable runs/animal/timepoint, *n* ≥ 19 (until 6 weeks), *n* ≥ 8 weeks 7–10, One-way analysis of variances (ANOVA) with repeated measurements to test for significant differences over time, and non-paired *t*-tests to test significance between groups.

Several gait parameters showed overall significant changes after autografting surgery. Mean hind paw print area was reduced to 33.6 ± 8.75 and 35.6 ± 9.83% (homotopic vs. heterotopic group) with no significant differences found between the two setup paradigms 1 week after injury. At the same time point duty cycle was reduced to 64.77 ± 10.6 and 69.09 ± 6.8% (homotopic vs. heterotopic groups) compared to the contralateral side. Swing time extended to 234.59 ± 55.6% (homotopic) and 225.96 ± 36% (heterotopic). Restoration of all three parameters to pre-injury levels was observed between postoperative weeks 7 and 10 with no significant differences between homotopic and heterotopic grafting (Figure 2). Furthermore, counting of retrograde labeled motoneurons innervating the femoral motor branch indicated similar numbers of reinnervating motoraxons (Supplementary Figure 2 and see Supplementary Data for methods).

#### Homotopic Grafting Results in Increased Wet Muscle Mass but Not in Improved Electrophysiological Parameters

**FIGURE 3:**
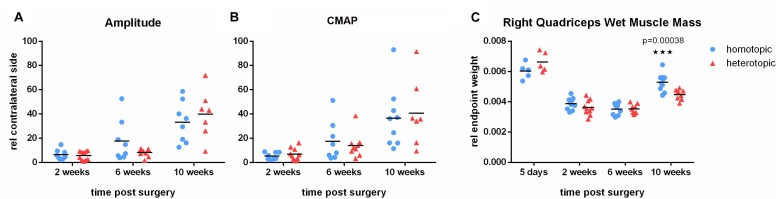
Electrophysiological evaluation and quantification of quadriceps muscle mass. **(A)** Peak amplitude of voltage signal as well as **(B)** evoked compound nerve action potential (CNAP) steadily increased over the observation period of 10 weeks. Homotopic grafting reached comparable outcome as heterotopic grafting. **(C)** Atrophy of the denervated quadriceps muscle is prominent until 6 weeks postoperatively. Functional reinnervation results in gain of muscle mass 10 weeks after injury. Homotopic grafting results in significantly higher muscle mass than heterotopic grafting. Mean, *n* = 5 (5 days), 8 (10 weeks heterotopic), 9 (6 weeks), 10 (2 weeks, 10 weeks homotopic), Multiple *t*-tests using Holm-Sidak correction for multiple comparison, (^∗∗∗^*p* < 0.001).

In order to further evaluate axonal regeneration through the phenotypically different autografts and reinnervation of the quadriceps muscle, we performed electrophysiological testing at 2, 6 and 10 weeks after homotopic or heterotopic femoral nerve autografting. Evoked CMAP and peak amplitude increased steadily over time indicating functional regeneration of motor axons and reinnervation of the denervated quadriceps muscle. As peak amplitude correlates well with the number of regenerated large (Aαß) myelinated fibers (Navarro, 2016), this demonstrates functional regeneration in both grafting groups over the time course of 10 weeks. However, both repair groups did not show full recovery of electrophysiological parameters (Figures 3A,B) during the observation period. Wet muscle mass of the quadriceps muscle evaluated 5 days, 2, 6 and 10 weeks after repair, demonstrated strong atrophy after femoral nerve defect in both autografting paradigms. Atrophy remained prominent until 6 weeks postoperatively. This was followed by increase in muscle mass on week 10. Interestingly, homotopic autografting of the femoral motor branch resulted in significantly higher quadriceps muscle mass recovery than heterotopic grafting (Figure 3C).

### Staggered Regeneration of Axons Occurs Independently of Pathway Modality and Graft Phenotype

**FIGURE 4:**
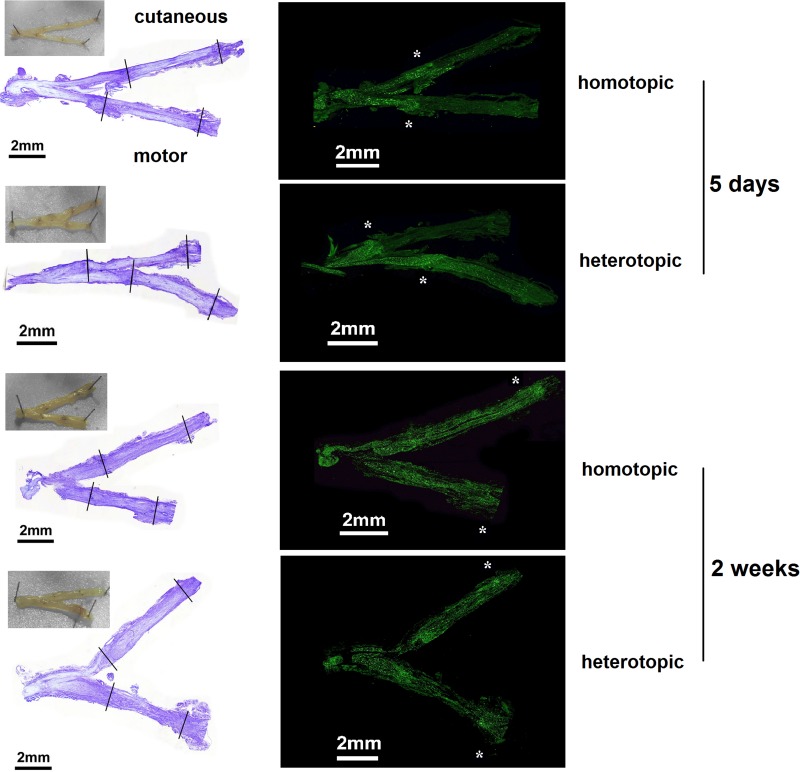
Representative examples of histological evaluation after homotopic/heterotopic nerve grafting. The proximal and distal coaptation sites in the motor as well as the cutaneous nerve can be seen in the macroscopic picture taken directly after excision. Suture sites are indicated in the cresyl violet overview (left panel). Axonal regeneration was visualized by Neurofilament 200 staining (right panel). Axonal regeneration front is staggered at the proximal coaptation site at 5 days (highlighted by asterisks). Two weeks after repair, axonal regeneration has surpassed the distal coaptation site in both grafting modalities.

To evaluate overall integrity of the grafts and axonal regeneration of the femoral motor and cutaneous branches, cresyl violet as well as neurofilament (NF) stainings were performed 5 days and 2 weeks after nerve repair. Representative images of macroscopical evaluation as well as cresyl violet staining showed intact suture sites in all excised samples (Figure 4). NF staining revealed the axonal regeneration front to be staggered at the proximal coaptation sites at 5 days. However, 2 weeks postoperatively, regenerated axons were visible in the distal parts of the motor and cutaneous branches regardless of homotopic or heterotopic grafting.

### Quantification of Baseline Gene Expression Analysis in Healthy Nerves

Quantitative reverse transcription PCR was used to evaluate expression profiles of 15 target genes over the motor and cutaneous central-peripheral axis of the femoral nerve. L2 and L3 dorsal (DR) and ventral roots (VR), the motor and cutaneous branches and a 10 mm piece of the proximal part of the healthy, contralateral femoral nerve were excised. Targets constitute of two well-described neurotrophic factors (BDNF and GDNF) and three of their receptors (Low affinity neurotrophic receptor – p75, Tropomyosin kinase receptor B – TrkB and GDNF receptor α1 – GFRα1), additionally we included the receptor of leukemia inhibitory factor (LIFR) as LIF is a known autocrine survival factor for Schwann cells (Ito et al., 1998; Dowsing et al., 1999). Furthermore, cell adhesion molecules, involved in axonal pathfinding (Fibronectin and its receptor integrin α5ß1) and in (re-)myelination processes (Cadm3, Cadm4, and L1CAM) were investigated. Finally Schwann cell specific glial fibrillary acidic protein (GFAP), proliferation associated protein Ki-67 and stress-responsive heat shock protein 70 (HSP70) were chosen to provide a more general overview of cell-status in the analyzed nerve samples. Comparison of expression levels of three different reference genes: HPRT, ß-Actin, and Ankrd27, was performed on a subset of samples. Expression levels of all three reference genes were proven to be unaffected by phenotypical and central-peripheral origin of nerve samples (Supplementary Figure 1). All subsequent data were normalized to HPRT.

#### Nerve Phenotype and Central-Peripheral Axis Do Not Affect Neurotrophic Factor Expression Whereas Their Receptors Are Strongly Affected

**FIGURE 5:**
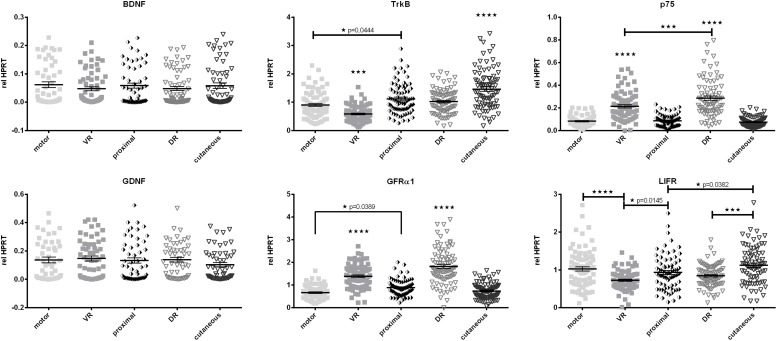
Comparison of baseline mRNA expression of neurotrophic factors and their receptors relative to HPRT in Schwann cellsof the femoral nerve constituents. In contrast to BDNF and GDNF, expression levels of neurotrophic factor receptors display significant discrepancies along the central-peripheral axis. motor, femoral nerve motor branch; cutaneous, femoral nerve cutaneous branch; proximal, mixed femoral nerve segment proximal to bifurcation; VR, ventral root; DR, dorsal root. Data are presented as mean ± SEM, *n* ≥ 57, One-way analysis of variances (ANOVA) with Tukey’s multiple comparison. (*p < 0.05, ^∗∗∗^*p* < 0.001, ${}^{*,*,**}$p < 0.0001).

Due to their potent effect on regeneration we evaluated the baseline mRNA levels of neurotrophic factors and their receptors (Figure 5). Brain derived neurotrophic factor (BDNF) as well as glial cell line-derived neurotrophic factor (GDNF) showed similar expression levels in dorsal, ventral roots as well as the peripheral mixed (proximal), motor, and cutaneous nerves. However, expression of the BDNF receptor tyrosine receptor kinase B (TrkB) was strongly increased in the cutaneous branch, when compared to other parts of the femoral nerve. The low-affinity neurotrophic factor receptor p75, as well as the GDNF receptor GFRα1, displayed central-peripheral commitment as both receptors showed significantly higher expression in the central L2, and L3 roots regardless of their phenotype. In contrast, LIFR demonstrated reduced expression in central parts of the femoral nerve.

#### Central-Peripheral Axis Strongly Affects Expression Levels of Cell Adhesion-Associated Genes

**FIGURE 6:**
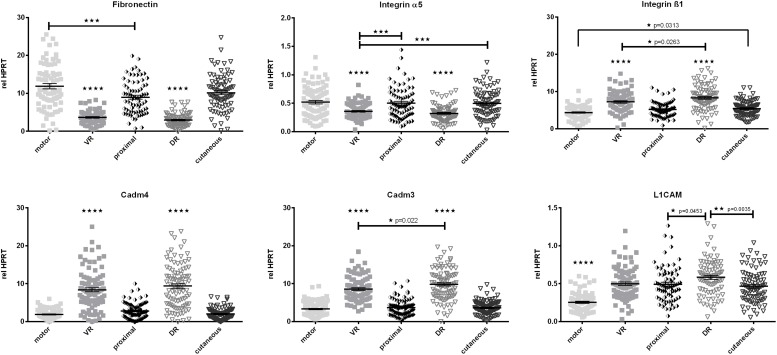
Quantification of baseline expression of cell adhesion molecules along the central-peripheral axis in healthy nerves. A strong spatial influence on expression of cell adhesion molecules was measured. Additionally the L1CAM mRNA levels in the femoral motor branch were significantly lower than in the central, proximal and cutaneous nerve segments. motor, femoral nerve motor branch; cutaneous, femoral nerve cutaneous branch; proximal, mixed femoral nerve segment proximal to bifurctaion; VR, ventral root; DR, dorsal root. Data are presented as mean ± SEM, *n* ≥ 57, One-way analysis of variances (ANOVA) with Tukey’s multiple comparison. (*p < 0.05, ^∗∗^*p* < 0.01, ^∗∗∗^*p* < 0.001, ${}^{*,*,**}$p < 0.0001).

Fibronectin and its neuronal integrin receptor α5ß1 play crucial roles in Schwann cell migration after peripheral nerve injury (de Luca et al., 2014). Also Cadm4, Cadm3, and the L1 cell adhesion molecule (L1CAM) are prominent factors in cell migration, axon pathfinding, and (re)-myelination processes. Therefore we evaluated differences in baseline expression of these factors along the different parts of the femoral nerve (Figure 6). All excised peripheral samples of the femoral nerve (proximal, cutaneous, and motor branch) showed higher expression of Fibronectin and Integrin α5 than the dorsal and ventral roots. However, expression levels of Integrin ß1, Cadm4, and Cadm3 were reduced in these samples when compared to the dorsal and ventral roots. L1CAM expression displayed a strong phenotypical/central discrepancy as the motor branch of the femoral nerve expressed significantly lower levels of L1CAM mRNA than the central roots and the proximal as well as cutaneous parts of the femoral nerve.

#### Cell Proliferation and Survival – Associated Genes Are Differentially Expressed Along the Central-Peripheral Axis

**FIGURE 7:**
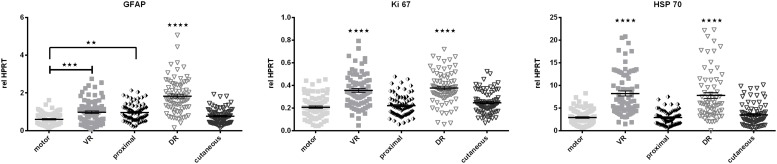
The central-peripheral axis in healthy nerves strongly influences the expression of GFAP, Ki-67, and HSP70 along the healthy femoral nerve. motor, femoral nerve motor branch; cutaneous, femoral nerve cutaneous branch; proximal, mixed femoral nerve segment proximal to bifurctaion; VR, ventral root; DR, dorsal root. Data are presented as mean ± SEM, *n* ≥ 57, One-way analysis of variances (ANOVA) with Tukey’s multiple comparison. (^∗∗^*p* < 0.01, ^∗∗∗^*p* < 0.001, ${}^{*,*,**}$p < 0.0001).

Cell proliferation-associated Ki-67 as well as stress responsive heat shock protein 70 (HSP70) show expression patterns similar

to Cadm3 and Cadm4 as well as Integrin ß1, GFRα1, and p75 as they exhibit higher mRNA levels in ventral and dorsal roots. Levels of Schwann cell specific cytoskeleton constituent, GFAP mRNA were highest in the L2 and L3 dorsal roots, and lowest in the motor branch of the femoral nerve (Figure 7).

### Spatiotemporal Expression Patterns After Homotopic and Heterotopic Autografting

We used qRT-PCR to evaluate the expression patterns of 15 regeneration-associated genes in a femoral nerve defect model. Target mRNAs were chosen upon literature research and include several mRNAs investigated in previous studies (see Table 2). Besides the investigation of expression of neurotrophic factors and their receptors we decided to include several cell adhesion molecules as well as proliferation and damage response – associated proteins. We evaluated gene expression patterns 5 days, 2, 6, and 10 weeks after nerve repair. The expression of these targets is first compared to the reference gene HPRT and then set into relation to the healthy contralateral corresponding femoral nerve part. We hypothesize that this broad spectrum of targets provides an intelligible overview of the cellular responses after nerve injury and repair with an either phenotypically matched or mismatched autologous nerve graft.

#### Expression Levels of BDNF and GDNF as Well as Their Receptors Are Strongly Affected by Injury and Axonal Regeneration

**FIGURE 8:**
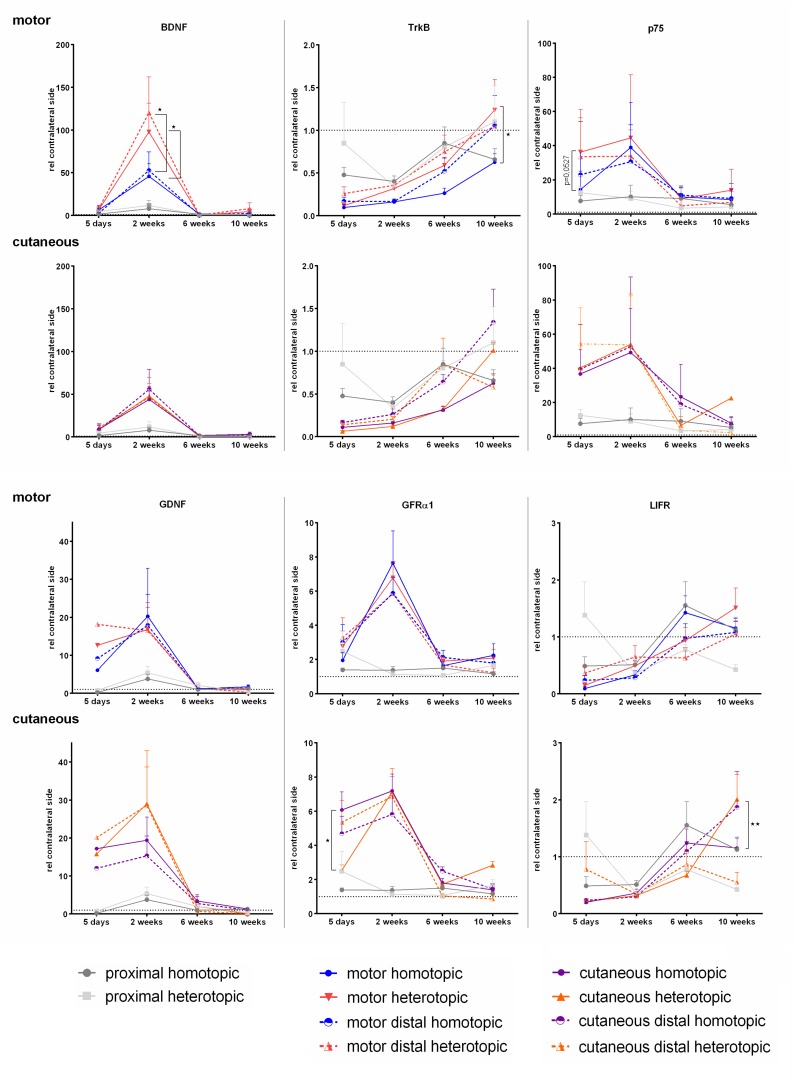
Normalized mRNA levels of neurotrophic factors and receptors after homotopic or heterotopic autografting relative to contralateral side. Data are presented as mean ± SEM, Two-way ANOVA with Tukey’s multiple comparison test. (*p < 0.05, ^∗∗^*p* < 0.01).

As neurotrophic factors and their signaling play crucial roles in axonal path finding and regeneration, Schwann cell proliferation as well as (re-) myelination, we investigated mRNA levels over time. The neurotrophic factors BDNF and GDNF were highly up regulated in the grafts, bridging the motor and cutaneous defects as well as the distal compartments of the respective branches (Figure 8). Two weeks after repair, expression of both factors reached a peak followed by normalization of mRNA levels at 6 and 10 weeks. Grafting of a purely sensory autologous transplant resulted in a significantly higher BDNF expression 2 weeks postoperatively in the graft bridging the motor defect as well as the distal motor branch, when compared to homotopic grafting. The phenotypical mismatch of a motor graft to bridge the defect of the cutaneous nerve did however, not influence BDNF expression. The phenotypically mixed femoral nerve fragment proximal to the injury displayed no up regulation of either neurotrophic factor at any timepoint.

Accordingly to previous studies (Boyd and Gordon, 2003a), we observed significant up regulation of the low affinity neurotrophic receptor p75 as soon as 5 days after nerve injury. Here a strong trend indicates higher expression in sensory derived graft in the motor defect than the phenotypically matched motor graft. We observed up regulation of p75 mRNA with peak expression at 2 weeks followed by normalization of p75 mRNA levels at later timepoints in all nerve samples distal to the injury. In contrast, the high affinity BDNF receptor TrkB is strongly down regulated after injury in motor as well as cutaneous pathways. Analogous to the preferential expression of BDNF in cutaneous nerves, the expression of TrkB is increased after heterotopic grafting in the motor branch on week 10.

The spatiotemporal expression pattern of GFRα1 closely resembles the GDNF expression pattern, with a peak expression at 2 weeks. However, in the denervated cutaneous branch the motor phenotype graft demonstrated significantly lower GFRα1 expression at 5 days than the phenotypically matched cutaneous graft. Similar to the neurotrophic factors and p75, the mRNA levels of GFRα1 reduced to contralateral, healthy levels over the time course of 6–10 weeks.

We were able to show a reduced expression of LIFR, 5 days, and 2 weeks after femoral nerve defect repair, with a steady increase to contralateral expression levels at 6 and 10 weeks. It is noteworthy, that at 10 weeks the motor derived graft in the cutaneous graft displayed higher levels of LIFR than the sensory derived autograft.

#### Expression of Fibronectin and Its Receptor Integrin α5ß1 Was Not Influenced by Autologous Nerve Grafting in Contrast to Cell Adhesion Molecules Cadm3, Cadm4, and L1CAM

**FIGURE 9:**
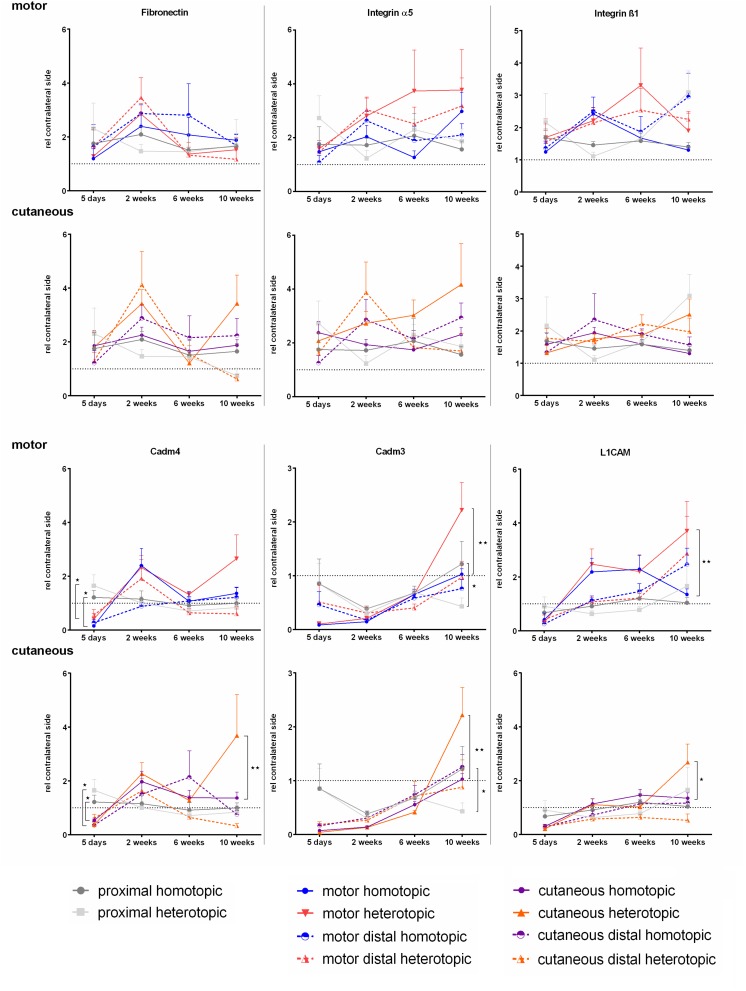
Normalized mRNA levels of cell adhesion molecules after homotopic and heterotopic autografting relative to contralateral side. Data are presented as mean ± SEM, Two-way ANOVA with Tukey’s multiple comparison test. (*p < 0.05, ^∗∗^*p* < 0.01).

We investigated the expression levels of Fibronectin mRNA as well as the spatiotemporal expression of integrins α5 and ß1, which form one of the major Fibronectin receptors. We did not observe any significant differences in either of the three mRNAs at any timepoint investigated. The levels in the injured nerves were similar to the contralateral side (Figure 9).

Schwann cell specific cell adhesion molecule Cadm4, as well as its heterophilic axonal counterpart Cadm3 were strongly down regulated 5 days after nerve grafting surgery in the repaired branches of the femoral nerve. This was followed by an up regulation of Cadm4 in the cutaneous and motor branch at 2 weeks. In contrast to Cadm4, Cadm3 mRNA levels stayed reduced for at least 2 weeks with a slow increase to contralateral levels at 10 weeks. Phenotypically mismatched grafting resulted in higher Cadm3 expression at 10 weeks in the respective grafts in the motor and the cutaneous femoral nerve.

Five days after nerve repair, both femoral nerve branches demonstrated reduced expression of L1CAM, whereas at 2 weeks, the mRNA levels normalized in all investigated nerve samples except for the grafts in the motor branch. Here an increase in expression was observed as well as higher expression of L1CAM in the grafted nerve segments at 10 weeks after heterotopic grafting compared to homotopic grafting.

#### Influence of Homotopic or Heterotopic Grafting on Expression of GFAP, Ki-67, and HSP70

**FIGURE 10:**
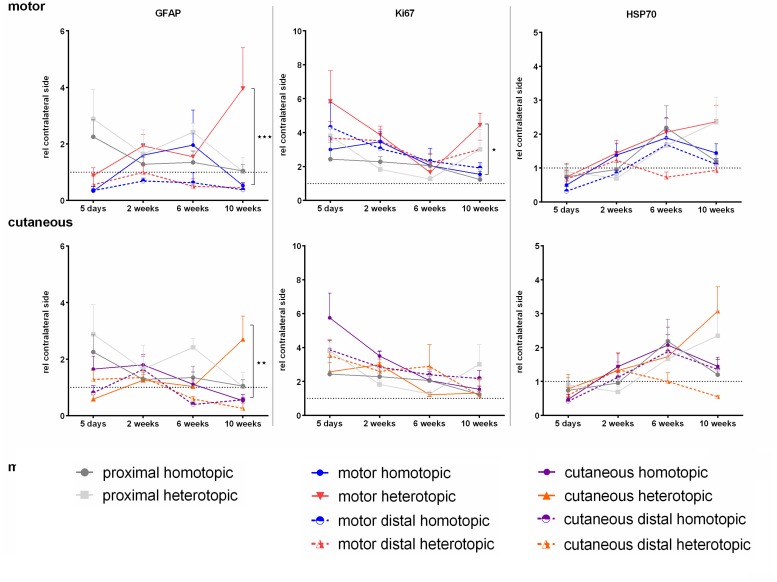
Normalized mRNA levels of GFAP, Ki-67, and HSP70 after homotopic and heterotopic autografting relative to contralateral side. Data are presented as mean ± SEM, Two-way ANOVA with Tukey’s multiple comparison test. (*p < 0.05, ^∗∗^*p* < 0.01, ^∗∗∗^*p* < 0.001).

In contrast to previously published data, we were not able to detect significant up regulation of GFAP in neither the motor nor the cutaneous branch after injury, except for a late increase in GFAP mRNA levels in the respective heterotopic grafts at 10 weeks (Figure 10). However, significantly enhanced expression of the proliferation-associated gene Ki-67 was observed at 5 days. The sensory graft demonstrated highest Ki-67 expression levels in both the cutaneous as well as the motor branch, indicating enhanced proliferation of Schwann cells of a sensory phenotype. Finally the chaperone HSP70 displayed a slight increase in expression over the time course of 10 weeks after injury, although no significant changes were determined.

## Discussion

### Experimental Model

We chose the femoral nerve model for our investigations, as it has been used to investigate phenotypical differences between motor and cutaneous pathways before (Brushart, 1990; Höke et al., 2006; Kawamura et al., 2010; Jesuraj et al., 2012; Brushart et al., 2013; Gordon and Borschel, 2017). Motor and cutaneous femoral branches are similar in size, fiber width and nerve cross sectional area (Brenner et al., 2006). Previous studies used ventral root isografts as phenotypically pure motor nerve grafts (Höke et al., 2006) or femoral nerve isografts from other animals (Moradzadeh et al., 2008; Kawamura et al., 2010). However, we adapted the model by performing homotopic and heterotopic autografting techniques for the repair of the motor and cutaneous branch (Figure 1). We used the femoral motor branch in contrast to the purely motor ventral root, as this more closely reflects the situation in other experimental autologous nerve transplantation models (i.e., the sciatic nerve defect model). Although a mixed nerve, a previous study by Marquardt and Sakiyama-Elbert proved motor phenotypical commitment of Schwann cells derived from the motor branch of the femoral nerve (Marquardt and Sakiyama-Elbert, 2015), deeming it a suitable graft for our purposes. Furthermore we performed autografting instead of isografting to represent clinical cases more accurately. Brushart et al. investigated the mRNA expression patterns of a variety of growth factors in cutaneous and motor nerves with differing denervation times ranging from 5 to 30 days (Brushart et al., 2013). Accordingly, we aimed to investigate the spatiotemporal gene expression pattern in denervated cutaneous and motor pathways, repaired with either phenotypically matched, or mismatched grafts.

### Functional Evaluation

So far, functional regeneration after injury to the femoral nerve was performed using single-frame motion analysis (SFMA) (Irintchev et al., 2005; Ahlborn et al., 2007). We identified three parameters directly correlating to de- and reinnervation of femoral nerve using gait analysis: Print area, swing time and duty cycle. Knee extension and bending are impaired after defect to the femoral nerve, as the quadriceps muscle is solely innervated by the femoral motor branch (Irintchev et al., 2005). The resulting abnormal bending of the knee joint results in abnormal plantar flexion and lifting of the heel, which decreases print area of the paw. The dynamic parameters swing time and duty cycle were also affected significantly by denervation of the quadriceps muscle, which is in accordance with findings after sciatic nerve injury in rats (Bozkurt et al., 2008), probably due to injury to the cutaneous branch of the femoral nerve (Puigdellívol-Sánchez et al., 2000; Kambiz et al., 2014). To the best of our knowledge this is the first study using catwalk automated gait analysis to evaluate regeneration in a femoral nerve defect model. Homotopic and heterotopic grafting resulted in comparable outcome regarding all three parameters. However, we consider the possibility that this evaluation method is lacking sensitivity due to the superior regenerative capacity of rodents combined with the short defect (6 mm) as well as the proximity to the target muscle and the provision of activated Schwann cells in both treatment paradigms.

Similarly, it was not possible to observe significant differences between the treatment groups in electrophysiological evaluations of the femoral motor branch. A general increase in CMAP and peak amplitude over time was determined. However, a large dispersion of values was apparent. The short defect of 6 mm in combination with high variability in the chosen functional tests is very likely hindering detection of differences between treatment groups. Previous studies claimed no difference after isografting of a femoral nerve defect with either a sensory or motor graft regarding histomorphometrical evaluations (Kawamura et al., 2010). Extended atrophy of the quadriceps muscle was measured until 6 weeks after injury, followed by an increase of muscle mass at 10 weeks in both treatment groups. Increase in muscle mass is only possible after successful reinnervation and reversal of atrophic processes of the quadriceps muscle. Interestingly, we measured significantly larger quadriceps muscle mass gain at 10 weeks after homotopic grafting than after heterotopic grafting (Figure 3C). This could indicate a superior capability of a phenotypically matched motor graft to regenerate motor axons when compared to a sensory graft, in accordance with data by Brenner et al. (2006) indicating inferior motor axonal regeneration through sensory grafts than size-matched motor grafts. As a growing body of literature has shown in numerous *in vitro* as well as *in vivo* studies, these effects could be explained by the phenotypical commitment of Schwann cells (Nichols et al., 2004; Höke et al., 2006; Moradzadeh et al., 2008; Jesuraj et al., 2012).

### Axonal Regeneration

In order to correlate functional regeneration and spatiotemporal gene expression patterns, we performed immunohistological evaluations of the repaired femoral nerves. NF staining revealed that 5 days after injury the main axonal regeneration front is still at the proximal coaptation site (Figure 4). Amongst other factors, the presence of a proximal suture site and ongoing clearance of residual myelin debris by macrophages hinder axonal reinnervation of the grafts in motor as well as cutaneous branches at this early timepoint (Fex Svennigsen and Dahlin, 2013). When confronted with a suture site, axons show staggered regeneration as a result of misalignment of Schwann cells in the fragmented extracellular matrix and asynchronous outgrowth of axons (Witzel et al., 2005). At 2 weeks we observed axonal reinnervation of all distal nerve stumps in both treatment groups, proving the capacity of phenotypically different Schwann cells to support axonal regeneration. Interestingly, Kawamura et al. (2010) observed no reinnervation of the distal branches 5 weeks after isografting of 10 mm nerve grafts in the same model, indicating a far slower regeneration of axons through isografts.

### Schwann Cell Phenotype and the Central-Peripheral Axis

Schwann cells are the glial cells of the peripheral nervous system and their phenotype is mainly associated with their functions as myelinating or non-myelinating cells, ensheathing Remak cells (Jessen and Mirsky, 2002). Schwann cells provide metabolic support for axons and are essential for axonal integrity (Nave, 2010; Harty and Monk, 2017). Over the last two decades, studies additionally revealed phenotypes of motor and sensory Schwann cells with distinct expression profiles for a variety of growth factors (Höke et al., 2006; He et al., 2012b; Jesuraj et al., 2012). We combined the expression profile data of more than 56 animals to investigate phenotypical commitment in healthy, contralateral nerves further. We focused on three sets of mRNAs: neurotrophic factors and their receptors, cell adhesion molecules and cell status-defining mRNAs, giving us insight into a broad spectrum of cellular responses.

Neurotrophic factors and their receptors play essential roles during development of the peripheral nervous system in axonal guidance, myelination and neuronal survival (Zhang et al., 2000; Wiese et al., 2001). The effects of neurotrophic factors and their receptors have been investigated intensively *in vitro* as well as *in vivo*. However, data on their baseline expression in healthy peripheral nerves is scarce. In contrast to Brushart et al. ( 2013) we did not observe significantly higher expression of neurotrophic factors BDNF or GDNF in L2 and L3 dorsal and ventral roots when compared to the peripheral motor, cutaneous or proximal segments of the healthy femoral nerve. The reason could be the use of HPRT instead of glycerinaldehyde-3-phosphat dehydrogenase (GAPDH) as a reference gene in the present study, as GAPDH is more highly expressed, resulting in generally very low relative expression levels in the studies by Höke et al. (2006), (Brushart et al., 2013). Furthermore higher variances were observed in the present study, as no pooling of samples was performed prior to qRT-PCR. In addition, we used a BDNF primer set, detecting all splicing variants of BDNF as expression of different splicing variants of BDNF is highly tissue dependent (Aid et al., 2007). Also, regarding BDNF expression our results are in line with data from Jesuraj et al. (2012), indicating no preferential expression along the central-peripheral axis nor in healthy phenotypically different Schwann cells. In stark contrast to neurotrophin expression levels, all investigated neurotrophin receptors showed large differences in expression along the central-peripheral axis in the healthy nerve.

We determined significantly increased TrkB expression in the cutaneous femoral branch and a very low relative expression of TrkB in ventral roots L2 and L3. This may indicate increased dependency of distal sensory Schwann cells on BDNF signaling including PI3K and the ras/ERK pathway (Boyd and Gordon, 2003b) or enhanced signaling of BDNF via the TrkB receptor rather than via p75. The low affinity neurotrophic factor p75 as well GFRα1 on the other hand were more highly expressed in dorsal and ventral roots, which could explain the responsiveness of motoneurons and their axons to introduced BDNF, and GDNF after ventral root avulsion (Pajenda et al., 2014). Additionally, a low expression is known in motoneurons and sensory neurons in adulthood (Ernfors et al., 1989; Wyatt et al., 1990). As p75 expression is correlating with proliferation (Provenzano et al., 2011), it was not surprising that Ki67 expression is also higher in central parts of the femoral nerve. However, the reason underlying the higher mitotic activity of dorsal and ventral root cells compared to peripheral segments remains target of further studies.

Schwann cells have the intrinsic capacity to produce not only LIF but also LIFR resulting in strong autocrine survival signaling. The lower expression of LIFR in central segments of the femoral nerve could be explained by the reduced relative numbers of non-neuronal cells in ventral and dorsal roots. This may also be the reason for our observation of lower expression of Fibronectin and integrin subunit α5 in ventral and dorsal roots. A close coregulation of Integrin α5 with Fibronectin can be expected, as the only binding partner for integrin α5 is Fibronectin. The interactions of neural crest cells and peripheral neurons with Fibronectin are mediated by integrins containing the ß1 subunit (Bronner-Fraser, 1985; Thiery and Duband, 1986; Plantman, 2013), which has numerous heterodimeric partners including vimentin, laminin, and L1CAM. Therefore an increased expression in proximity to neuronal cell bodies is not surprising. Dorsal root ganglions as well as motoneurons express especially high levels of integrin ß1 as reviewed by Plantman (Plantman, 2013). Furthermore, He et al. (2012b) demonstrated, that integrin ß1 is more highly expressed in sensory nerves, which is in line with our observation, that dorsal roots express significantly higher levels of ß1 than ventral roots. The same holds true for the cutaneous femoral branch exhibits higher expression than the motor branch. Another class of cell adhesion molecules, also known as nectin-like molecules are Cadm4 and Cadm3. Cadm4 is mainly expressed in Schwann cells and builds strong heterophilic interactions with Cadm3, which is expressed by axons (Maurel et al., 2007). In the healthy nerve both molecules are clustered at the periaxonal membrane and at the internodes and Schmidt-Lanterman incisures of myelinated axons (Maurel et al., 2007; Perlin and Talbot, 2007; Spiegel et al., 2007). Cadm4 and Cadm3 are essential for myelination, therefore the increased central expression observed in this study can be explained by the high numbers of myelinated axons in L2 and L3 ventral roots (Biscoe et al., 1982) and the substantial expression in dorsal root ganglions (Chen et al., 2016).

The neuronal cell adhesion molecule L1CAM is part of the Ig superfamily of cell adhesion molecules and displays homophilic as well as heterophilic (i.e., NCAM) interactions. We detected less pronounced expression levels of L1CAM in the motor femoral branch in healthy nerves than in the cutaneous branch. This is in accordance with other studies, where sensory Schwann cells exhibit higher levels of L1CAM than motor Schwann cells *in vitro* (He et al., 2012a) and *in vivo* (Jesuraj et al., 2012). However, this phenomenon is restricted to the peripheral nerve segments, as we observed similar expression levels of L1CAM in purely motor ventral roots, and dorsal roots. GFAP is known to be mainly expressed in non-myelinating or repair Schwann cells, explaining the low expression in femoral motor nerve branch. The higher expression in ventral and dorsal roots could be due to a higher proportion of undifferentiated, proliferating Schwann cells in these segments, as we also detected enhanced expression of proliferation-associated Ki67 and stress responsive HSP70 levels in ventral and dorsal roots. Additionally the significantly higher expression of GFAP in dorsal roots is expected, as they contain a high number of non-myelinating Schwann cells. HSP70 is one of the earliest up regulated proteins after nerve injury in zebrafish (Nagashima et al., 2011). This might occur because ventral and dorsal roots are more prone to experience stress-inducing traction and compression even in healthy animals, as they do not possess a protective connective tissue layer.

### Spatiotemporal Expression Patterns After Homotopic and Heterotopic Grafting

Brain derived neurotrophic factor is a potent survival factor for motoneurons after proximal axotomy (Kishino et al., 1997; Pajenda et al., 2014). Furthermore, its expression is necessary for remyelination (Zhang et al., 2000) and as a guidance molecule for axonal regeneration (Boyd and Gordon, 2003a). In our study, grafting of a purely sensory autologous transplant resulted in a significantly higher BDNF expression 2 weeks postoperatively in the graft bridging the motor defect as well as the distal motor branch, when compared to homotopic grafting. This indicates not only that Schwann cells of a sensory phenotype express higher levels of BDNF when they encounter motor axons, but also influence expression levels in the distal motor branch. Sensory Schwann cells enhance expression of BDNF, probably to overcome their reduced capacity to support motor axonal regeneration. Sensory axons are less susceptible to phenotypical mismatch than motor axons, therefore no compensation in the cutaneous graft was observed (Marquardt and Sakiyama-Elbert, 2015). BDNF signals via two receptors, TrkB and p75.

Denervation of motor and sensory branches of the femoral nerve resulted in a marked decrease of TrkB, which is consistent with data provided by Funakoshi et al. (1993). Over the time course of 6–10 weeks normalization of TrkB expression was observed, which allows normal remyelination of regenerated axons (Cosgaya et al., 2002). We observed up regulation of p75 mRNA with peak expression at 2 weeks followed by normalization of p75 mRNA levels at later timepoints in all nerve samples distal to the injury. This is consistent with data from previous work which indicate a role in pre-myelination (Cosgaya et al., 2002). Although the general up regulation of p75 after injury is well described (Boyd and Gordon, 2003b; Cragnolini and Friedman, 2008; Jessen and Mirsky, 2016), the exact spatiotemporal expression pattern in phenotypically different, injured nerves has not been described earlier. Timeline of expression levels closely resembles the BDNF mRNA expression pattern indicating co-regulation of BDNF and its low affinity receptor p75. Binding of BDNF to p75 results in activation of the Ras/ERK pathway and activation of c-Jun, which in turn activates BDNF as well as GDNF transcription (Arthur-Farraj et al., 2012; Jessen and Mirsky, 2016).

GDNF is produced by myelinating and non-myelinating Schwann cells (Xu et al., 2013) and in our setup, exhibited a close resemblance in expression pattern to its high affinity receptor GFRα1, with a peak expression at 2 weeks. GDNF signaling may occur in a ret-dependent and ret-independent way. Both signaling modalities promote cell survival and, as Schwann cells are the main producers of GDNF, it is thought that the elevated levels of GDNF in the denervated parts of the femoral nerve branches induce strong pro-survival cues via GFRα1 in an autocrine way. Interestingly, homotopic grafting in the cutaneous branch resulted in a delayed up regulation of GFRα1 mRNA.

Leukemia inhibitory factor receptor (LIFR) mRNA is known to be reduced after crush and transection injury, however, the expression pattern after autologous nerve grafting as well as the influence of phenotypically mismatched Schwann cells has not been investigated. As LIF expression is up regulated after peripheral nerve injury (Banner and Patterson, 1994), observed down regulation of LIFR for at least 2 weeks may prevent non-neuronal cells from undesired consumption of LIF (Ito et al., 1998). In contrast to previously published data we did not observe a significant increase in mRNA levels of neither Fibronectin nor its heterodimeric integrin receptor α5ß1. A possible explanation could be post-transcriptional regulation of these molecules.

Cell adhesion molecules Cadm4, which is mainly expressed on Schwann cells as well as its heterophilic axonal counterpart Cadm3, were strongly down regulated 5 days after nerve grafting surgery in the repaired branches of the femoral nerve (Figure 9). In contrast to Zelano et al. (2009) we did not observe Cadm4 up regulation 5 days after injury, which might originate from the different animal model used, as they investigated a sciatic nerve defect without repair. However, we observed up regulation of Cadm4 in the cutaneous and motor branch at 2 weeks, the time point at which axonal reinnervation of the nerve grafts and even the distal segments is taking place. This is in line with the role Cadm4 plays in remyelination processes. It is required for myelination and its expression is increased by axonal contact (Perlin and Talbot, 2007; Spiegel et al., 2007). More precisely, Cadm4 induces clustering of Cadm3 on axons to enable efficient heterophilic interaction between regrown axons and resident Schwann cells. This also explains the spatiotemporal expression of Cadm3, which is reaching normal levels between 6 and 10 weeks after injury. The significantly higher expression of Cadm3 in sensory-derived grafts in both branches indicates ongoing reorganization processes of myelination in phenotypically mismatched grafts. As uninjured Schwann cells also express Cadm3 another explanation could be the re-established expression of Cadm3 by Schwann cells at this late timepoint after injury (Zelano et al., 2009). Finally, Cadm3 has been shown to inhibit myelination via activation of PI3 Kinase/Akt signaling. This may result in less myelination of regenerated axons in these grafts (Chen et al., 2016).

The neuronal cell adhesion molecule L1CAM plays a role in early myelination and its expression coincides with a decrease in proliferation (Guseva et al., 2009; Lutz et al., 2016). L1CAM signaling results in activation of the ERK pathway and induces axonal growth (Maness and Schachner, 2007). The restoration of L1CAM expression 2 weeks after injury and the observed axonal regeneration at this timepoint confirms this observation. In accordance with Qian-ru et al we measured higher L1CAM mRNA levels in the grafts bridging the motor defect than in the cutaneous defect. An increase in L1CAM to induce axonal growth in the motor branch is to be expected, considering that the baseline expression of L1CAM in motor nerves is lower than in cutaneous nerves (Jesuraj et al., 2012; He et al., 2016). We did not observe significant influences of phenotypical mismatch at early stages after nerve grafting.

Furthermore we discovered a decrease of GFAP mRNA in the denervated motor branch 5 days after injury, which indicates regulation of GFAP expression on the protein level, as other groups provided evidence for enhanced levels of GFAP after nerve defect (Triolo, 2006; Wang et al., 2010). Heterotopic grafting however, resulted in increased levels of GFAP in the respective grafts at late stages of regeneration, indicating higher numbers of non-myelinating Schwann cells in phenotypically mismatched grafts (Yang and Wang, 2015). Proliferation of denervated Schwann cells is a hallmark of the repair phenotype (Jessen and Mirsky, 2016). We observed slightly increased expression of Ki67 in sensory derived grafts, providing further evidence for a higher proliferative capacity of cutaneous Schwann cells (Jesuraj et al., 2014).

**FIGURE 11:**
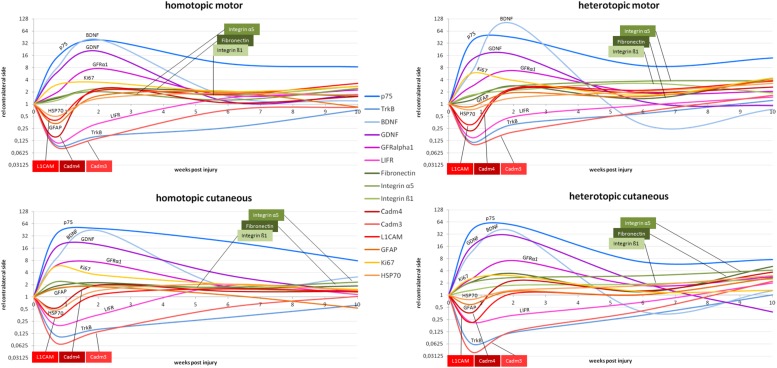
Schematic overview of the mRNA expression levels relative to uninjured contralateral nerves. Upper row depicts the temporal expression patterns in the graft bridging the motor defect. The lower row shows the temporal expression patterns in the graft bridging the cutaneous defect. On the left hand side, the phenotypically matched homotopic graft is depicted, whereas the right hand side shows the temporal expression in the phenotypically mismatched graft in the respective nerves.

We summarized spatiotemporal expression patterns in a comprehensive overview of all investigated genes in motor as well as sensory grafts in Figure 11.

## Conclusion

Sensory nerve autografts are the clinical gold standard to bridge peripheral nerve defects. However, functional outcome after autologous nerve transplantation is often unsatisfying (Meek et al., 2005; Secer et al., 2008; Yang et al., 2011). In this study we investigated the effect of phenotypical commitment of peripheral motor and sensory nerve grafts on axonal regeneration in a rat femoral nerve defect model. Furthermore, we examined the influence of motor and sensory phenotype on the expression level of several regeneration-associated genes in healthy and injured femoral nerves. We uncovered highly significant differences in gene expression along the central-peripheral axis and between motor and sensory constituents of the healthy femoral nerve. A significant influence of phenotypical mismatch on the spatiotemporal expression of several investigated mRNAs in regenerating motor and cutaneous nerves was evident. Overall, we observed similar but not identical spatiotemporal expression of a variety of regeneration-associated genes in motor (mixed) and cutaneous grafts. Furthermore, we elucidated that phenotypical mismatched grafting can influence expression patterns of distal nerve segments, thereby potentially influencing axonal regeneration, and target reinnervation. This study shows that in a small 6 mm femoral nerve defect the graft phenotype has only a minor influence on functional regeneration. Success of axonal regeneration after nerve defect repair is highly dependent on a tightly regulated spatiotemporal profile of genes. The differences observed in our study might prove to be crucial in large nerve defects, where mismatched Schwann cell phenotype may interfere with correct path finding of motor and sensory nerves to their respective end organs. However, the effect of phenotypically mismatched long grafts on axonal regeneration and functional recovery -especially after delayed repair- remains to be elucidated.

## Ethics Statement

This study was carried out according to Austrian law and the guide for the care and use of laboratory animals, and was approved by the City Government of Vienna.

## Author Contributions

DH contributed to conception and all evaluations as well as the analyses and wrote the manuscript. MK performed the surgeries. JH performed the gait analysis. CS contributed to critical revision of data and manuscript. MS contributed in establishment of qRT-PCR protocols. LG performed the histological evaluations. RS and TH contributed to conception. HR, AN, and AH contributed to conception and critically reviewed the manuscript.

## Conflict of Interest Statement

The authors declare that the research was conducted in the absence of any commercial or financial relationships that could be construed as a potential conflict of interest.
