# Supplementary figures and images for: Spatiotemporal Differences in Gene Expression Between Motor and Sensory Autografts and Their Effect on Femoral Nerve Regeneration in the Rat
Source: Front Cell Neurosci. 2019 May 8;13:182. doi: 10.3389/fncel.2019.00182 (PMC6519304; doi:10.3389/fncel.2019.00182)

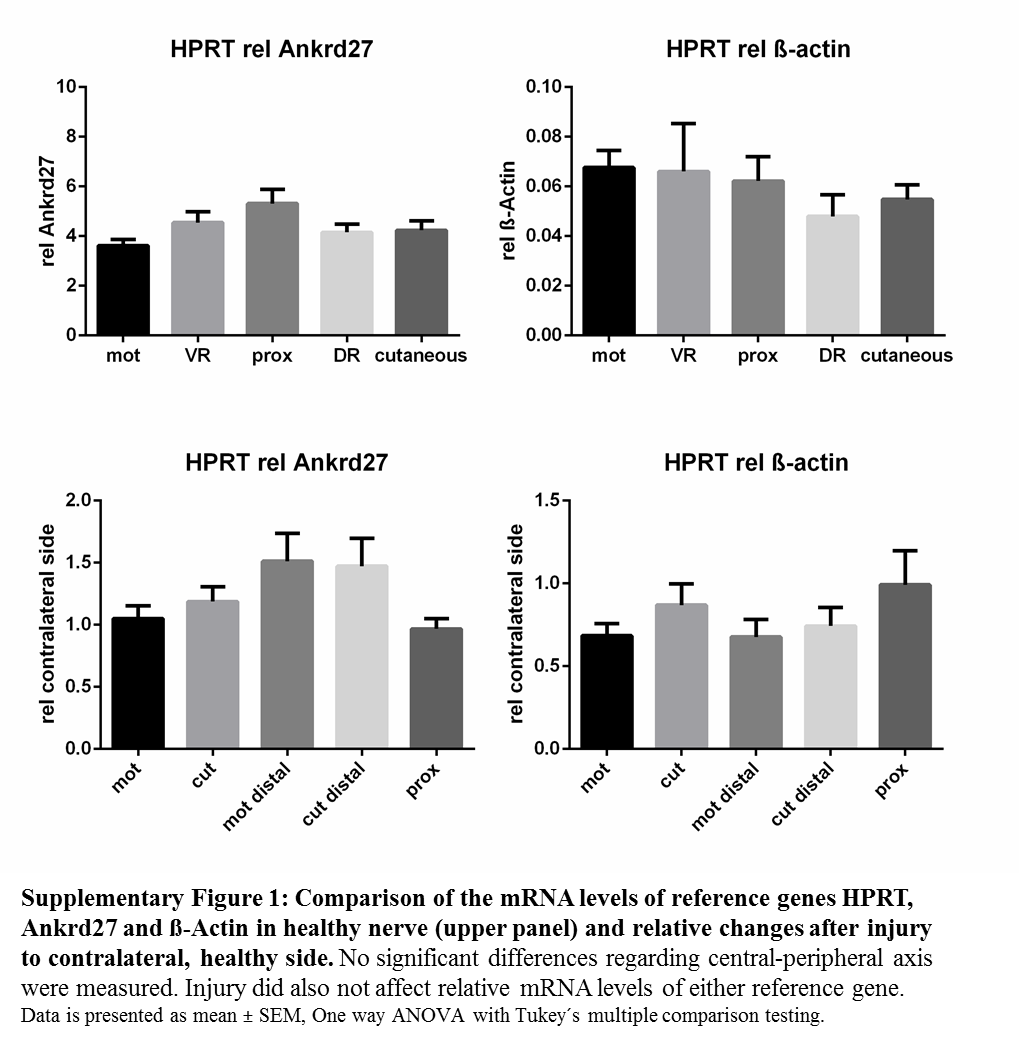

Supplement: Supplementary file 1 [file Image_1.TIF]

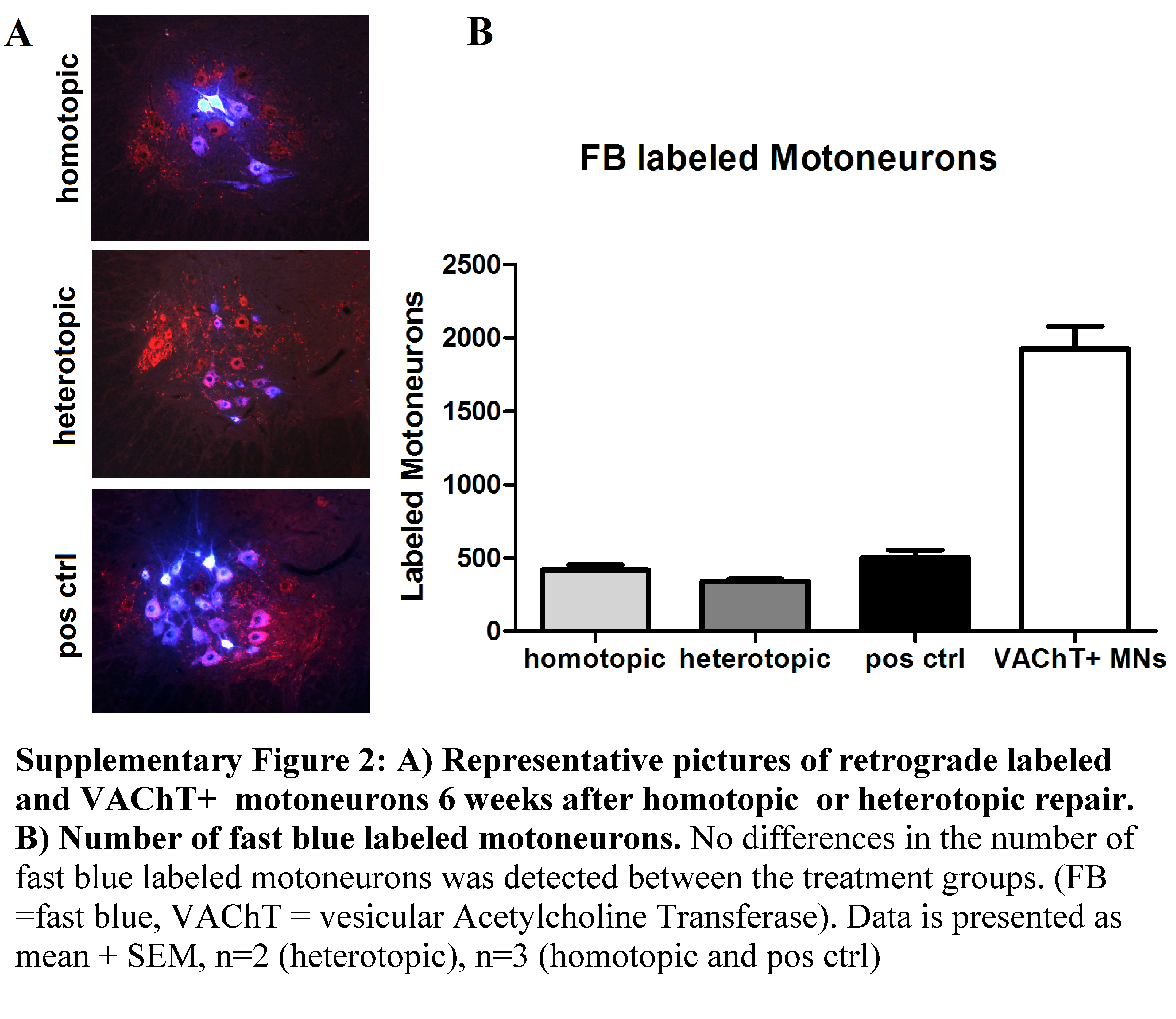

Supplement: Supplementary file 2 [file Image_2.TIF]
