# Supplementary material for: Spatiotemporal Differences in Gene Expression Between Motor and Sensory Autografts and Their Effect on Femoral Nerve Regeneration in the Rat
Source: Front Cell Neurosci. 2019 May 8;13:182. doi: 10.3389/fncel.2019.00182 (PMC6519304; doi:10.3389/fncel.2019.00182)
Supplement: Supplementary file 3 [file Table_1.DOCX]

**Supplementary methods**

**qRT-PCR**

RNA-isolation was performed in an RNAse-free environment. Snap-frozen samples were homogenized using D-Stroy-Sticks (Biozym, GER) and RNA was isolated using peqGOLD TriFast (VWR, USA). Yield and purity were checked by photometry at A260/A280 for each sample and integrity was checked using agarose gel electrophoresis by random sampling. 1µg DNAseI-digested (Promega, USA) RNA was used for subsequent cDNA synthesis according to manufacturer´s instructions (EasyScript™ RTase, ABM, CAN)

Analysis of mRNA levels were performed in technical triplicates by qRT-PCR using 18 ng cDNA, primer according to table 2 (Microsynth, SUI) and KAPA SYBR (KAPA SYBR, Peqlab/PerfeCTa SYBR, VWR, USA) on a Bio-Rad CFX 96 cycler (Bio-Rad, CA, USA).

qRT-PCR protocol was as follows:

1. 95°C for 3:00 minutes
2. 95°C for 0:03 to 0:10
3. Primer Annealing Temperature for 0:30 + Read plate
4. 72°C for 0:10 + Read plate
5. Go to step 2 for 49 more times
6. 95°C for 0:10
7. Primer Annealing Temperature for 0:30
8. Melting curve from Primer Annealing Temperature to 95°C, increment 0.5°C, 0:05 + Read plate

Raw data evaluation was performed using BioRad CFX Manager 3.1.

**Retrograde labeling**

Operated animals of the 6 week survival groups (n=2-3 / group) and healthy control animals (n = 3) were deeply reanesthetized as described above. The femoral nerve was exposed and cut distally of the distal coaptation site of the motor branch. The stump of the nerve was covered with few crystals of Fast Blue (FB, Illing Plastics GmbH, Breuberg, Germany) - wound closure and postsurgical analgesia as described above. Five days after the application of the fluorescent dye the animals were reanesthetized and perfused transcardially with 4% paraformaldehyde in 0.1 M phosphate buffer (pH = 7.4).

**Immunohistochemistry**

Twenty-five μm thick transverse spinal cord sections were cut on a cryostat (Leica CM1850, Leica GmbH, Germany) and mounted onto gelatin-coated glass slides. Nonspecific binding sites were subsequently blocked with 1% milk powder solution. Primary antibodies were incubated overnight at 4 °C, washed, and then incubated with fluorescent-conjugated secondary antibodies for 1 h at roomtemperature. For vesicular choline-acetyltransferase (VChAT) immunohistochemistry samples were preincubated in 3% normal goat serum for 1 h, then incubated with a rabbit anti-VAChT polyclonal antibody (Synaptic System, Goettingen, Germany; 1:400 - 139103) overnight at 4 °C, followed by washing and anti-rabbit Alexa Fluor 546 secondary antibody (Thermo Fisher Scientific [Life Technologies], Waltham, MA, USA, 1:400 - A11010) for 1.5 h at room temperature. Fluorescent signals were detected in an Olympus FX51 epifluorescence microscope equipped with a DP70 digital camera (Olympus Ltd, Tokyo, Japan). Confocal microscopic images were obtained by using an Olympus FluoView® FV10i compact confocal microscope. Digital images were resized and their contrast and brightness were adjusted.

**Cell counts**

The number of retrogradely labeled and VChAT positive cells was determined (Fig. 1E). To avoid double counting of the same neuron present in two consecutive sections, the retrogradely labeled neurons were mapped with the aid of an Olympus (Olympus Ltd, Tokyo, Japan) drawing tube, and their locations were compared to those of labeled neurones in the previous section (Nógrádi et al., 2007; Pintér et al., 2010). The proportion of FB+/VChAT+ cells was determined in each experimental group. All sections from the L2-L3 motoneuron pool were used.
